# Cardiac Surgery Morbidity and Mortality in Hypertensive and Arrhythmic Patients: A Retrospective Analysis

**DOI:** 10.7759/cureus.48505

**Published:** 2023-11-08

**Authors:** Abdullah Abdulrahman Bayazed, Abdullah Khalid Alassiri, Abdullah Alaa Farid, Muhannad Salem Dawood, Khalid Mohammed Alshuqayfi, Abdulaziz Mustafa Adnan, Faisal Othman BinJahlan, Turki Bader Aljohani, Ragab Shehata Debis, Khalid E Al-Ebrahim

**Affiliations:** 1 Faculty of Medicine, King Abdulaziz University, Jeddah, SAU; 2 Cardiac Surgery Unit, Department of Surgery, King Abdulaziz University Hospital, King Abdulaziz University, Jeddah, SAU

**Keywords:** postoperative complications, coronary artery bypass surgery, atrial fibrillation, arrhythmias, hypertension

## Abstract

Background: Hypertension (HTN) is the most significant modifiable risk factor for cardiovascular disease (CVD) and overall mortality. HTN is defined as a systolic blood pressure of ≥140 mmHg and/or a diastolic blood pressure of ≥ 90 mmHg. Generally, arrhythmias are characterized by a disruption of the heart's regular rhythm. They are strongly associated with increased risks of CVDs and sudden death. The most common arrhythmia worldwide is atrial fibrillation (AF). HTN, diabetes mellitus (DM), and coronary artery disease (CAD) are major risk factors for arrhythmias.

Objective: We aimed to identify the postoperative effects and risk factors of HTN and cardiac arrhythmia in patients who underwent cardiac surgery at King Abdulaziz University Hospital (KAUH) from 2015 to 2022.

Methods: A retrospective record review was conducted by collecting data from KAUH electronic medical records. A total of 402 patients participated in this study. This study includes all hypertensive and arrhythmic patients who underwent cardiac surgeries.

Results: Of the 402 patients studied, 209 had pre-operative HTN, and 47 had preoperative AF. Developing post-operative arrhythmia was found to significantly increase perioperative morbidity and mortality (p < 0.001). Risk factors for HTN and arrhythmia included increased age, higher BMI, and DM.

Conclusion: The findings of this study suggest an association between preoperative HTN and AF and elevated rates of postoperative morbidity and mortality. AF emerged as the predominant arrhythmia type. It is advisable to optimize patients' health status prior to surgical procedures. Moreover, further research is recommended in this field to deepen our understanding of the perioperative implications of HTN and arrhythmias.

## Introduction

Hypertension (HTN) is the most significant modifiable risk factor for cardiovascular disease (CVD) and overall mortality [1,2]. A systolic blood pressure (SBP) of ≥ 140 mmHg and/or diastolic blood pressure (DBP) ≥ 90 mmHg is the definition of high blood pressure [3]. HTN prevalence is increasing worldwide [3], and in Saudi Arabia, in 2014, according to the Saudi Ministry of Health, the prevalence of HTN was 15.1% [4]. Arrhythmias are a common finding with HTN [5].

Generally, arrhythmias are characterized by a disruption of the heart’s regular rhythm. They are strongly associated with increased risks of cardiovascular issues and lead to disability, mortality, higher healthcare costs, and lower quality of life [6-11]. Some types of cardiac arrhythmias include atrial fibrillation (AF), atrial flutter, supraventricular tachycardia (SVT), ventricular fibrillation, ventricular tachycardia, complete heart block, and premature ventricular contractions (PVC) [12]. The most common arrhythmia worldwide is AF [13]. High blood pressure, diabetes mellitus (DM), and coronary artery disease are major risk factors [12].

Many previous studies have been conducted on HTN and arrhythmia. A remarkable result is that up to 90% of patients with AF who engaged in large randomized clinical outcome trials of new anticoagulant or antiarrhythmic medications for the treatment of AF had a history of HTN [14]. A study done in Liverpool in 2021 found that hypertensive patients had a significant risk for postoperative AF [15]. In Saudi Arabia, two studies conducted in 2019 found that cardiac arrhythmia is related to HTN and coronary artery diseases [13,16].

No previous studies were conducted at King Abdulaziz University Hospital (KAUH), Saudi Arabia, sharing the postoperative complications and mortality rates in hypertensive and arrhythmic patients. As a result, the primary objective of our study was to discern and quantify the morbidity and mortality rates within the cohort of patients who underwent cardiac surgery at our institution between 2015 and 2022.

## Materials and methods

This study aims to identify the development of morbidity and mortality due to cardiac arrhythmia and HTN as outcomes in patients who have undergone cardiac surgeries in KAUH in Jeddah, Saudi Arabia (KSA), between 2015 and 2022. The study also determines the risk factors associated with HTN and arrhythmia. The ethical committee at KAUH has provided its approval for this study, and the reference number is 244-22.

Participants and settings

A total of 402 patients participated in this study. The study was conducted in the Cardiac Surgery Department in June 2022 at KAUH, a tertiary governmental center in Jeddah, Saudi Arabia. The data that were collected were from 2015 to 2022. Our study included all hypertensive and arrhythmic patients who had undergone cardiac surgeries. We included all patients who were 14 years of age or older and were treated at the Cardiac Surgery Department in KAUH and who were hypertensive and/or arrhythmic. The patient’s data were gathered retrospectively from the KAUH medical records.

Study design

A retrospective record view was conducted by collecting data from KAUH electronic medical records and transferring them to Excel sheets. The information that we collected included the following: patients' medical records; age; gender; diagnosis; body mass index (BMI); preoperative AF; preoperative and postoperative blood pressures; preoperative potassium (k+) and magnesium (Mg2+) levels; preoperative ejection fraction (EF); preoperative creatinine levels; number of HTN medications taken by the patient; whether the patient was diabetic, dyslipidemic, obese, or had a history of previous stroke; which operation was done; postoperative blood pressures; development of arrhythmias postsurgery; number of days in the hospital postoperatively; and postoperative mortality and morbidity.

Definitions

Postoperative arrhythmic conditions encompass patients who have exhibited any of the following cardiac irregularities after surgery: AF, atrial flutter, heart block, PVC, SVT, ventricular fibrillation, or ventricular tachycardia.

Morbidity is characterized by the development of any postoperative complications, including but not limited to acute kidney injury (AKI), hemothorax, hemorrhage, infection, pyrexia, thrombophlebitis, cardiac arrest, colonic distention, heart blockage, myocardial infarction (MI), shock, or ventricular tachycardia.

Data entry and analysis

We used Statistical Product and Service Solutions (SPSS, version 26) (IBM SPSS Statistics for Windows, Armonk, NY) for statistical analysis. The incidence was calculated as a percentage with a 95% confidence interval (CI), and the significance level was set at P = 0.05. We applied the chi-squared test (χ2) to qualitative data that were expressed as numbers and percentages to examine the relationships among the variables. We used the Mann-Whitney test to analyze nonparametric variables and presented quantitative data as mean and standard deviation (mean ± SD). To compute the odds ratio, a 95% CI was used to assess the risk factors of HTN, arrhythmia, and AF. Statistical significance was defined as a P-value of less than 0.05.

## Results

Hypertension

The mean age of studied patients was 53.27 ± 12.47 years, and 88.1% were males. The mean BMI, body surface area (BSA), and HTN medications used were 28.13 ± 23.32 kg/m^2^, 1.8 ± 0.19, and 2.13 ± 0.72 drugs, respectively. Of patients, 73.9%, 15.7%, 19.9%, and 3.7% had diabetes mellitus (DM), dyslipidemia, obesity, and stroke, respectively. Preoperative AF was present among 3.7% of patients, and 11.7% had arrhythmia. Patients who had HTN had a significantly older mean age, a higher mean BMI, and a lower BSA value (P = < 0.05). At the same time, hypertensive patients had a lower mean number of HTN drugs used (P = < 0.05). HTN was significantly higher among females, diabetics, those having no dyslipidemia, and overweight patients (P = < 0.05), as seen in Table 1.

**Table 1:** Baseline characteristics and differences between hypertensive and nonhypertensive patients according to their demographics, body mass index, body surface area, HTN medications, and comorbidities. Body mass index (BMI), body surface area (BSA), hypertension (HTN), atrial fibrillation (AF), premature ventricular contractions (PVC), and supraventricular tachycardia (SVT)

Variable	Mean of total (402)	Mean of HTN (209)	Mean of No HTN (No.: 193)	χ2	P-value
Age	53.27 ± 12.47	58.23 ± 10.34	47.89 ± 12.3	8.15*	< 0.001
BMI	28.13 ± 23.32	29.89 ± 32.12	26.22 ± 3.36	3.14*	0.002
BSA	1.8 ± 0.19	1.85 ± 0.2	1.75 ± 0.15	5.38*	< 0.001
HTN medications	2.13 ± 0.72	2.01 ± 0.63	2.25 ± 0.8	3.92*	< 0.001
Operation number	3.39 ± 0.81	3.48 ± 0.77	3.32 ± 0.84	2.45	0.014
Gender					
Male	354 (88.1)	177 (84.7)	177 (91.7)	4.7	0.03
Female	48 (11.9)	32 (15.3)	16 (8.3)		
DM					
No	105 (26.1)	70 (33.5)	35 (18.1)	12.26	< 0.001
Yes	297 (73.9)	139 (66.5)	158 (81.9)		
Dyslipidemia					
No	339 (84.3)	164 (87.5)	175 (90.7)	11.3	0.001
Yes	63 (15.7)	45 (21.5)	18 (9.3)		
Obesity					
No	186 (46.3)	68 (32.5)	118 (61.1)	33.07	< 0.001
Overweight	136 (33.8)	88 (42.1)	48 (24.9)		
Obese	80 (19.9)	53 (25.4)	27 (14)		
Stroke					
No	387 (86.3)	200 (95.7)	187 (96.9)	0.4	0.527
Yes	15 (3.7)	9 (4.3)	6 (3.1)		
Preoperative AF					
No	387 (86.3)	201 (96.2)	186 (96.4)	0.01	0.915
Yes	15 (3.7)	8 (3.8)	7 (93.6)		
Postoperative arrhythmia					
No	355 (88.3)	26 (13.5)	21 (10)	1.13	0.286
Yes	47 (11.7)	167 (86.5)	188 (90)		
If yes, what type: (No. 47)					
Not mentioned	1 (2.1)	0 (0.0)	1 (0.5)	6.72	0.566
Atrial fibrillation	16 (34.3)	8 (4.1)	8 (3.8)		
Atrial flutter	1 (2.1)	0 (0.0)	1 (0.5)		
Heart block	9 (19.1)	5 (2.6)	4 (1.9)		
PVC	2 (4.2)	1 (0.5)	1 (0.5)		
SVT	1 (2.1)	0 (0.0)	1 (90.5)		
Ventricular fibrillation	9 (19.1)	6 (3.1)	3 (1.4)		
Ventricular tachycardia	8 (17)	6 (3.1)	2 (1)		

As for the preoperative data, the mean SBP, DBP, and mean blood pressure (BP) were 136.5 ± 21.01, 82.17 ± 13.26, and 95.73 ± 10.94 mmHg, respectively. The mean K+ and Mg2+ (mmol/L) were 4.13 ± 1.93 and 0.98 ± 0.27, respectively. The mean ejection fraction (%) was 48.27 ± 9.67%, and the mean creatinine was 106.17 ± 94.72. According to the operative data, the mean CBP time was 123.2 ± 39.2, and the mean cross-clamp time was 78.76 ± 27.61. Postoperatively, the mean SBP was 115.15 ± 16.22, the mean DBP was 66.45 ± 12.17, and the mean duration of hospital stay was 14.36 ± 8.53 days. Of patients, 11.2% had morbidities, with bleeding (6.7%) the most common. Regarding outcomes, 23 patients (5.7%) died. Patients who had HTN had a significantly higher mean preoperative SBP, DBP, and overall mean BP (P = < 0.05). At the same time, hypertensive patients had a lower mean level of K+ (mmol/L) or Mg2+ (mmol/L) (P = < 0.05). Regarding intraoperative data, patients who had HTN had a significantly lower mean CBP time and lower mean ACX time than patients without HTN (P = < 0.05). Hypertensive patients also had higher mean postoperative SBP and DBP, but a shorter mean duration of hospital stay than nonhypertensive patients (P = < 0.05). A nonsignificant difference was found between hypertensive and nonhypertensive patients regarding the occurrence of morbidity or mortality (P = > 0.05). However, hypertensive patients had a significantly higher percentage of bleeding (P = < 0.05), as shown in Table 2.

**Table 2:** Difference between hypertensive and nonhypertensive patients according to their preoperative, intraoperative, and postoperative data; morbidity; and mortality. Systolic blood pressure (SBP), diastolic blood pressure (DBP), blood pressure (BP), acute kidney injury (AKI), myocardial infarction (MI) N.B. * = Mann-Whitney test

Variable	Mean of the Total (402)	Mean of HTN (209)	Mean of No HTN (No. 193)	Test	P-value
Preoperative data					
SBP	136.5 ± 21.01	150.96 ± 18.1	120.83 ± 9.61	17.33*	< 0.001
DBP	82.17 ± 13.26	91.45± 11.51	72.13 ± 5.36	17.2*	< 0.001
Mean BP	95.73 ± 10.94	106.01 ± 4.3	86.74 ± 5.65	5.73*	< 0.001
K+ (mmol/L)	4.13 ± 1.93	4.1 ± 0.42	4.16 ± 2.77	4.36*	< 0.001
Mg2+ (mmol/L)	0.98± 0.27	0.88 ± 0.18	1.08 ± 0.3	5.67*	< 0.001
Ejection fraction (%)	48.27 ± 9.67	48.2 ± 10.56	48.34 ± 8.65	0.24*	0.806
Pre-operative creatinine	106.17 ± 94.72	105.48 ± 93.03	106.92 ± 96.76	1.96*	0.049
Intraoperative data					
Cardiopulmonary bypass time	123.2 ± 39.2	120.26 ± 37.59	126.38 ± 40.73	2.39*	0.017
Cross-clamp time	78.76 ± 27.61	73.98 ± 25.99	83.94 ± 28.43	4.18*	< 0.001
Postoperative data					
SBP	115.15 ± 16.22	120.62 ± 16.64	109.56 ± 13.72	7.3*	< 0.001
DBP	66.45± 12.17	67.97 ± 13.34	64.89 ± 10.65	2.11*	0.035
Hospital stays (days)	14.36 ± 8.53	13.21 ± 9.43	15.63 ± 7.23	5.38*	< 0.001
Morbidity					
No	357 (88.8)	182 (87.1)	175 (90.7)	1.3	0.254
Yes	45 (11.2)	27 (12.9)	18 (9.3)		
If yes, specify: (No. 45)					
AKI	3 (0.7)	1 (0.5)	2 (1)	0.42	0.516
Hemothorax	1 (0.2)	0 (0.0)	1 (0.5)	1.08	0.297
Bleeding	27 (6.7)	20 (9.6)	7 (3.6)	5.65	0.017
Infection	4 (1)	3 (1.4)	1 (0.5)	0.85	0.355
Fever	1 (0.2)	1 (0.5)	0 (0.0)	0.92	0.336
Thrombophlebitis	1 (0.2)	0 (0.0)	1 (0.5)	1.08	0.297
Cardiac arrest	4 (1)	1 (0.5)	3 (1.6)	1.17	0.178
Colon distension	1 (0.2)	0 (0.0)	1 (0.5)	1.08	0.297
Heart blockage	1 (0.2)	0 (0.0)	1 (0.5)	1.08	0.297
MI	1 (0.2)	0 (0.0)	1 (0.5)	1.08	0.297
Shock	2 (0.5)	0 (0.0)	2 (1)	2.17	0.14
Ventricular tachycardia	1 (0.2)	1 (0.5)	0 (0.0)	0.92	0.336
Mortality					
No	379 (94.3)	198 (94.7)	181 (93.8)	0.16	0.681
Yes	23 (5.7)	11 (5.3)	12 (6.2)		

We conducted a multivariate logistic regression analysis to assess the risk factors (independent predictors) of HTN among studied patients. We found that being older, having DM, and having a higher mean SBP or DBP were risk factors (independent predictors) of HTN, as shown in Table 3.

**Table 3:** Multivariate logistic regression analysis of risk factors of HTN among studied patients. Body surface area (BSA), hypertension (HTN), diabetes mellitus (DM), systolic blood pressure (SBP), diastolic blood pressure (DBP), blood pressure (BP)

Variable	Odds Ratio (CI: 95%)	P-value
Age	0.98 (1.12–2.34)	0.023
BSA	0.4 (0.87–1.09)	0.325
HTN medications	0.98 (0.04–1.10)	0.237
Operation number	0.19 (0.2–0.182)	0.156
Gender	1.23 (0.91–2.7)	0.768
DM	0.98 (1.03–2.37)	0.004
Dyslipidemia	0.2 (0.13–1.87)	0.354
Obesity	0.02 (0.14–1.34)	0.341
Preoperative data		
SBP	0.14 (0.95–1.09)	0.007
DBP	0.94 (1.36– 2.64)	0.018
Mean BP	0.92 (0.31– 2.12)	0.06
K+ (mmol/L)	0.31 (0.73–1.92)	0.165
Mg2+ (mmol/L)	0.12 (0.17– 0.98)	0.71
Intraoperative data		
Cardiopulmonary bypass time	0.06 (0.37–0.98)	0.112
Cross-clamp time	0.5 (0.1–0.74)	0.09
Postoperative data		
SBP	0.2 (0.53–1.07)	0.135
DBP	0.18 (0.731.98)	0.082

Arrhythmia

Table 4 shows that patients with arrhythmia had a significantly higher mean age, a higher mean BMI, and a higher mean BSA but a lower mean number of HTN medications (P = < 0.05). At the same time, a significantly higher percentage of arrhythmia patients presented with obesity, stroke, or preoperative AF (P = < 0.05).

**Table 4:** Baseline characteristics and differences between patients with and without arrhythmia according to their demographics, BMI, BSA, HTN medications, and comorbidities. Body surface area (BSA), hypertension (HTN), diabetes mellitus (DM), systolic blood pressure (SBP), diastolic blood pressure (DBP), blood pressure (BP)

Variable	Arrhythmia (No. 47)	No arrhythmias (No. 355)	χ2	P-value
Age	60.04 ± 8.77	52.37 ± 12.58	3.83	< 0.001
BMI	27.67 ± 67.19	26.87 ± 4.01	2.14	0.032
BSA	1.87 ± 0.18	1.79 ± 0.18	2.87	0.004
HTN medications	1.83 ± 0.76	2.17 ± 0.71	2.79	0.005
Operation number	3.25 ± 0.89	3.41 ± 0.8	1.31	0.189
Gender				
Female	6 (12.8)	42 (11.8)	0.03	0.853
Male	41 (87.2)	313 (88.2)		
DM				
No	11 (23.4)	94 (26.5)	0.2	0.652
Yes	36 (76.6)	261 (73.5)		
Dyslipidemia				
No	40 (85.1)	299 (84.2)	0.02	0.876
Yes	7 (14.9)	56 (15.8)		
Obesity				
Normal	13 (27.7)	173 (48.7)	8.52	0.014
Overweight	19 (40.4)	117 (33)		
Obese	15 (31.9)_	65 (18.3)		
Stroke				
No	42 (89.4)	345 (97.2)	7.06	0.008
Yes	5 (10.6)	10 (2.8)		
Preoperative AF				
No	41 (87.2)	346 (97.5)	12.09	0.001
Yes	6 (12.8)	9 (2.5)		

Table 5 shows that arrhythmia patients had a significantly lower preoperative mean SBP, a lower preoperative mean EF (%), a lower mean postoperative DBP, and a lower mean hospital stay in days (P = < 0.05). Arrhythmia patients also had a significantly higher percentage of comorbidities, such as AKI, hemothorax, bleeding, cardiac arrest, heart block, MI, or ventricular tachycardia compared with patients with no arrhythmia (P = < 0.05).

**Table 5:** Difference between patients with and without arrhythmia according to their preoperative, intraoperative, and postoperative data; morbidity; and mortality. Systolic blood pressure (SBP), diastolic blood pressure (DBP), blood pressure (BP), acute kidney injury (AKI), myocardial infarction (MI) N.B. * = Mann-Whitney test

Variable	Arrhythmia (No. 47)	No arrhythmia (No. 355)	Test	P-value
Preoperative data				
SBP	129.15 ± 15.19	137.47 ± 21.49	2.76	0.0061
DBP	78.65 ± 9.92	82.64 ± 13.58	1.37	0.168
Mean BP	95.73 ± 10.94	98.75 ± 12.34	0.3	0.243
K+ (mmol/L)	4.04 ± 0.54	4.14 ± 2.05	0.55	0.578
Mg2+ (mmol/L)	0.95 ± 0.28	0.98 ± 0.26	0.88	0.376
Ejection fraction (%)	44.61 ± 11.42	48.77 ± 9.31	2.35	0.019
Pre-operative creatinine	100.58 ± 46.91	106.91 ± 99.37	0.83	0.403
Intraoperative data				
Cardiopulmonary bypass time	130.3 ± 56.43	122.26 ± 36.32	0.53	0.593
Cross-clamp time	75.98 ± 33.45	79.13 ± 26.78	1.02	0.305
Postoperative data				
SBP	114.64 ± 17.43	115.22 ± 16.07	0.2	0.834
DBP	64.2 ± 14.57	66.76 ± 11.8	2.08	0.038
Hospital stay (days)	12 ± 8.56	14.68 ± 8.49	3.39	0.001
Morbidity				
No	33 (70.2)	324 (91.3)	18.5	< 0.001
Yes	14 (29.8)	31 (8.7)		
If yes, specify: (No. 45)				
AKI	2 (4.3)	1 (0.3)	8.48	0.003
Hemothorax	1 (2.1)	0 (0.0)	7.57	0.006
Bleeding	7 (14.9)	20 (5.6)	5.68	0.017
Infection	0 (0.0)	4 (1.1)	0.53	0.465
Fever	0 (0.0)	1 (0.3)	0.13	0.716
Thrombophlebitis	0 (0.0)	1 (0.3)	0.13	0.716
Cardiac arrest	2 (4.3)	2 (0.6)	5.74	0.017
Colon distension	0 (0.0)	1 (0.3)	0.13	0.716
Heart block	1 (2.1)	0 (0.0)	7.57	0.006
MI	1 (2.1)	0 (0.0)	7.57	0.006
Shock	1 (2.1)	1 (0.3)	2.85	0.091
Ventricular tachycardia	1 (2.1)	0 (0.0)	7.57	0.006
Mortality				
No	36 (76.6)	343 (96.6)	30.85	< 0.001
Yes	11 (23.4)	12 (3.4)		

While conducting the multivariate logistic regression analysis to assess the risk of arrhythmia among studied patients, we observed that having a higher mean age or a higher mean BSA were risk factors (independent predictors) of having arrhythmia, as seen in Table 6.

**Table 6:** Multivariate logistic regression analysis of risk factors of arrhythmia among studied patients. Body surface area (BSA), hypertension (HTN), diabetes mellitus (DM), systolic blood pressure (SBP), diastolic blood pressure (DBP), blood pressure (BP)

Variable	Odds ratio (CI:95%)	P-value
Age	1.13 (0.94–2.34)	0.001
BSA	1.8 (0.8–3.24)	0.015
HTN medications	1.27 (0.77–2.09)	0.344
Operation number	0.19 (0.78–1.8)	0.405
Gender	0.48 (0.51–2.27)	0.462
DM	0.46 (0.23–1.6)	0.899
Dyslipidemia	0.8 (0.31–1.2)	0.646
Obesity	0.91 (0.58–1.43)	0.689
Preoperative data		
SBP	1.02 (0.99–1.05)	0.176
DBP	1.02 (0.97–1.07)	0.342
Mean BP	0.3 (0.5–1.07)	0.918
K+ (mmol/L)	1.04 (0.44–2.42)	0.927
Mg2+ (mmol/L)	0.37 (0.08–1.94)	0.191
Intraoperative data		
Cardiopulmonary bypass time	0.99 (0.97–1)	0.138
Cross-clamp time	1 (0.98–1.03)	0.404
Postoperative data		
SBP	0.5 (0.13–1.5)	0.915
DBP	0.1 (0.97–1.04)	0.433

## Discussion

This study is a comparative analysis of the incidence of morbidity and mortality as a function of preoperative HTN and arrhythmias in cardio-surgery patients at KAUH in Jeddah. The data suggest that the incidence of postoperative morbidity and mortality was not significantly higher in patients with preoperative HTN but significantly higher in patients with preoperative arrhythmia. Preoperative HTN and arrhythmia were, however, associated with a significantly shorter mean duration of hospital stay postsurgery and a significantly greater risk of postoperative bleeding. Logistic regression analysis found advanced age and BSA to be significant predictors of arrhythmias and advanced age and DM as risk factors for HTN.

Similar to our study, expert opinion in an analysis by Sanders et al. reported the lack of enough evidence to support the hypothesis that lower preoperative blood pressure was associated with lower perioperative morbidity and mortality [17]. Another cohort study, however, found an increased risk of postoperative mortality with preoperative diastolic HTN, whereas the association between systolic HTN and postoperative mortality was insignificant, quite similar to our results [18]. A trial conducted by Karimi et al. reported preoperative HTN and arrhythmia as predictors of morbidity and complications after cardiac surgery, which is consistent with our results [19]. The association observed in our study between preoperative AF and mortality following cardiac surgery is also evident in the available literature [20,21].

This study showed bleeding to be the most common postoperative complication, which is consistent with a study by Pahwa et al., showing 47.3% of subjects requiring postoperative blood product transfusion and 3.3% requiring reoperation for bleeding [22]. Our patients were hypertensive, and the majority were type 2 diabetics, which could play a role in the occurrence of postoperative bleeding, corroborated by another cohort study [23]. Other studies also showed chronic HTN among the factors associated with excessive bleeding after cardiac surgery [24,25]. This emphasizes HTN as an important prevalent risk factor for bleeding complications in our study population.

According to our results, bleeding is a significantly prevalent postsurgical complication in patients with preoperative arrhythmia as well. Studies have shown an association between a higher risk of bleeding and stroke in arrhythmic patients [26]. The significant bleeding risk, shown in Table 2 and Table 4, can also be explained by the coexistence of preoperative HTN and AF in patients [27]. Our study did not report any significant risk of postoperative AKI, stroke, or arrhythmias in hypertensive patients, which does not correlate with the available literature [28-30].

A single case of postsurgical hemothorax in both hypertensive and arrhythmic patients was reported, which may be due to arterial rupture following pulmonary artery catheter placement [31]. Our study shows a significant prevalence of postsurgical arrhythmias and renal and cardiac failure in arrhythmic patients, which can be explained by conduction abnormalities, thrombo-embolic events, and hemodynamic compromise [32-34].

Our outcomes from multivariate logistic regression analysis identified older age as a risk factor for both HTN and arrhythmias. This is evident from previous studies that determined advancing age to be an independent risk factor for HTN, particularly SBP and arrhythmias [35,36]. DM is another risk factor significantly associated with HTN but insignificantly associated with arrhythmia. Previous studies show that, due to increased peripheral vascular resistance in diabetics, their risk of HTN is doubled [37]. The available evidence, however, contradicts the latter outcome and reports diabetes to be a significant predictor of arrhythmias [30,38]. This can be explained by diabetes’ association with atrial and ventricular remodeling and molecular and autonomic malfunction of the heart [38]. The outcomes in the available literature reported BMI and BSA as risk factors for both HTN and arrhythmia, only the latter of which is consistent with our analysis [39,40]. Contrary to our results, it is evident that dyslipidemia predisposes patients to both HTN and arrhythmia [41,42].

Because surgical complications such as bleeding, AKI, and sternal wound complications can trigger arrhythmia, every effort should be made to avoid them by implementing specific and strict surgical policies and guidelines [25,33,43,44].

A significant proportion of our hypertensive population had dyslipidemia [41], diabetes [37], and obesity [39], and a significant proportion of our arrhythmic population had stroke [26,32] and obesity [40]. AF was the most prevalent type of arrhythmia [45]. The prevalence of HTN was significantly higher in the female population [46], which is consistent with general demographic cardiology trends. The data reveal an indirect relation between hypertensive medication and arrhythmia because some HTN medications reduce AF, as supported by previous studies [47].

This is the only known study of its kind that is designed as a comparative analysis to estimate the effects of preoperative HTN and arrhythmia on perioperative complications and mortality in cardiac patients in a single trial. Previous studies consider either morbidity only or mortality only [19,20]. Thus, the strength of our study lies in its outcome size and comparative design.

Limitations

Our study is not devoid of limitations. First, it adhered to a retrospective record review design, which, by its nature, carries inherent limitations. The potential for selection bias arises from the fact that the study was conducted exclusively within a single medical center. Furthermore, the study's single-center scope, centered in Jeddah, Saudi Arabia, restricts the generalizability of its findings to a broader population. It is noteworthy that the study period coincided with the COVID-19 pandemic, which led to a notable decrease in the number of surgeries, not only at our center but potentially impacting our sample size. Therefore, we advocate for future investigations with larger sample sizes. These identified limitations underscore the necessity for further research in this field, with an emphasis on enhancing external validity, enabling healthcare settings to make informed adjustments based on evidence.

## Conclusions

This study aimed to determine postoperative morbidity and mortality rates due to cardiac arrhythmia and HTN in cardiac surgery patients at KAUH in Jeddah between the years 2015 and 2022. The results indicate that preoperative HTN and arrhythmia were more or less associated with higher postoperative morbidity and mortality. The most common type of arrhythmia was found to be AF. These results showed that the development of HTN and arrhythmia are associated with advanced age, BMI or BSA, and DM. It is recommended that the patient’s status be optimized before surgery and that more research be conducted in this field to further understand the perioperative effects of HTN and arrhythmias.

## References

[1] Stanaway JD, Afshin A, Gakidou E (2018). Global, regional, and national comparative risk assessment of 84 behavioural, environmental and occupational, and metabolic risks or clusters of risks for 195 countries and territories, 1990-2017: a systematic analysis for the Global Burden of Disease Study 2017. Lancet.

[2] Roth GA, Abate D, Abate KA (2018). Global, regional, and national age-sex-specific mortality for 282 causes of death in 195 countries and territories, 1980-2017: a systematic analysis for the Global Burden of Disease Study 2017. Lancet.

[3] Mills KT, Bundy JD, Kelly TN (2016). Global disparities of hypertension prevalence and control: a systematic analysis of population-based studies from 90 countries. Circulation.

[4] Al-Ebrahim EK, Madani TA, Al-Ebrahim KE (2022). Future of cardiac surgery, introducing the interventional surgeon. J Card Surg.

[5] Alebrahim K, Shafei H, Tahir M (1995). Cardiac surgery in the Kingdom of Saudi Arabia. Asian Cardiovasc Thorac Ann.

[6] Murakoshi N, Aonuma K (2013). Epidemiology of arrhythmias and sudden cardiac death in Asia. Circ J.

[7] Fazzini PF, Marchi F, Pucci P (1976). Prognostic value of intraventricular conduction blocks in acute myocardial infarction. Acta Cardiol.

[8] Napodano RJ, Kalra T (1978). Complete right bundle branch block and acute myocardial infarction an ominous sign. J Fam Pract.

[9] Col JJ, Weinberg SL (1972). The incidence and mortality of intraventricular conduction defects in acute myocardial infarction. Am J Cardiol.

[10] Hollander G, Nadiminti V, Lichstein E, Greengart A, Sanders M (1983). Bundle branch block in acute myocardial infarction. Am Heart J.

[11] Rizzon P, Di Biase M, Baissus C (1974). Intraventricular conduction defects in acute myocardial infarction. Br Heart J.

[12] Clay-Weinfeld K, Callans M (2019). Common postcardiothoracic surgery arrhythmias. Crit Care Nurs Clin North Am.

[13] Mashat AA, Subki AH, Bakhaider MA (2019). Atrial fibrillation: risk factors and comorbidities in a tertiary center in Jeddah, Saudi Arabia. Int J Gen Med.

[14] Manolis AJ, Rosei EA, Coca A (2012). Hypertension and atrial fibrillation: diagnostic approach, prevention and treatment. Position paper of the working group ‘hypertension arrhythmias and thrombosis’ of the European Society of Hypertension. J Hypertens.

[15] Qureshi M, Ahmed A, Massie V, Marshall E, Harky A (2021). Determinants of atrial fibrillation after cardiac surgery. Rev Cardiovasc Med.

[16] Ibrahim N, Ph K, Alghalayini Alghalayini, Milyani A, Neyas A, Alturkistani R, Niazi R (2019). Conduction abnormalities: types, associated factors and their effect on clinical outcomes of patients admitted to the coronary care unit at King Abdulaziz University Hospital. Saudi J Intern Med.

[17] Sanders RD, Hughes F, Shaw A (2019). Perioperative quality initiative consensus statement on preoperative blood pressure, risk and outcomes for elective surgery. Br J Anaesth.

[18] Venkatesan S, Myles PR, Manning HJ (2017). Cohort study of preoperative blood pressure and risk of 30-day mortality after elective non-cardiac surgery. Br J Anaesth.

[19] Karimi A, Ahmadi H, Davoodi S (2008). Factors affecting postoperative morbidity and mortality in isolated coronary artery bypass graft surgery. Surg Today.

[20] dos Santos CA, Oliveira MA, Brandi AC (2014). Risk factors for mortality of patients undergoing coronary artery bypass graft surgery. Rev Bras Cir Cardiovasc.

[21] Cadore MP, Guaragna JC, Anacker JF (2010). A score proposal to evaluate surgical risk in patients submitted to myocardial revascularization surgery. Rev Bras Cir Cardiovasc.

[22] Pahwa S, Bernabei A, Schaff H (2021). Impact of postoperative complications after cardiac surgery on long-term survival. J Card Surg.

[23] Pereira KM, de Assis CS, Cintra HN (2019). Factors associated with the increased bleeding in the postoperative period of cardiac surgery: a cohort study. J Clin Nurs.

[24] Lopes CT, Brunori EF, Cavalcante AM, Moorhead SA, Swanson E, Lopes Jde L, de Barros AL (2016). Factors associated with excessive bleeding after cardiac surgery: a prospective cohort study. Heart Lung.

[25] Elassal AA, Al-Ebrahim KE, Debis RS (2021). Re-exploration for bleeding after cardiac surgery: revaluation of urgency and factors promoting low rate. J Cardiothorac Surg.

[26] Redfors B, Gray WA, Lee RJ, Ellenbogen KA, Bonafede M, Ben-Yehuda O (2017). Patients with atrial fibrillation who are not on anticoagulant treatment due to increased bleeding risk are common and have a high risk of stroke. JACC Clin Electrophysiol.

[27] Aronow WS (2017). Hypertension associated with atrial fibrillation. Ann Transl Med.

[28] Thanavaro J, Taylor J, Vitt L, Guignon MS (2019). Predictors and outcomes of acute kidney injury after cardiac surgery. Nephrol Nurs J.

[29] Healey JS, Connolly SJ (2003). Atrial fibrillation: hypertension as a causative agent, risk factor for complications, and potential therapeutic target. Am J Cardiol.

[30] Yamashita K, Hu N, Ranjan R, Selzman CH, Dosdall DJ (2019). Clinical risk factors for postoperative atrial fibrillation among patients after cardiac surgery. Thorac Cardiovasc Surg.

[31] Tanner TG, Colvin MO (2020). Pulmonary complications of cardiac surgery. Lung.

[32] Capucci A, Villani GQ, Aschieri D (1997). Risk of complications of atrial fibrillation. Pacing Clin Electrophysiol.

[33] Alghamdi AA, Aqeeli MO, Alshammari FK, Altalhi SM, Bajebair AM, Al-Ebrahim Frcsc KE (2022). Cardiac surgery-associated acute kidney injury (CSA-Aki) in adults and pediatrics; prevention is the optimal management. Heart Surg Forum.

[34] Uhm JS, Shim J, Wi J (2014). First-degree atrioventricular block is associated with advanced atrioventricular block, atrial fibrillation and left ventricular dysfunction in patients with hypertension. J Hypertens.

[35] Pinto E (2007). Blood pressure and ageing. Postgrad Med J.

[36] Curtis AB, Karki R, Hattoum A, Sharma UC (2018). Arrhythmias in patients ≥80 years of age: pathophysiology, management, and outcomes. J Am Coll Cardiol.

[37] Epstein M, Sowers JR (1992). Diabetes mellitus and hypertension. Hypertension.

[38] Grisanti LA (2018). Diabetes and arrhythmias: pathophysiology, mechanisms and therapeutic outcomes. Front Physiol.

[39] Jiang SZ, Lu W, Zong XF, Ruan HY, Liu Y (2016). Obesity and hypertension. Exp Ther Med.

[40] Vyas V, Lambiase P (2019). Obesity and atrial fibrillation: epidemiology, pathophysiology and novel therapeutic opportunities. Arrhythm Electrophysiol Rev.

[41] Otsuka T, Takada H, Nishiyama Y, Kodani E, Saiki Y, Kato K, Kawada T (2016). Dyslipidemia and the risk of developing hypertension in a working-age male population. J Am Heart Assoc.

[42] Li ZZ, Du X, Guo XY (2018). Association between blood lipid profiles and atrial fibrillation: a case-control study. Med Sci Monit.

[43] Alebrahim K, Al-Ebrahim E (2020). Prevention, classification and management review of deep sternal wound infection. Heart Surg Forum.

[44] Almramhi K, Aljehani M, Bamuflih M (2022). Frequency and risk factors of unplanned 30-day readmission after open heart surgeries: a retrospective study in a tertiary care center. Heart Surg Forum.

[45] Aronow WS, Ahn C, Gutstein H (1996). Prevalence of atrial fibrillation and association of atrial fibrillation with prior and new thromboembolic stroke in older patients. J Am Geriatr Soc.

[46] Frye RL, Higgins MW, Beller GA (1988). Major demographic and epidemiologic trends affecting adult cardiology. J Am Coll Cardiol.

[47] Healey JS, Baranchuk A, Crystal E, Morillo CA, Garfinkle M, Yusuf S, Connolly SJ (2005). Prevention of atrial fibrillation with angiotensin-converting enzyme inhibitors and angiotensin receptor blockers: a meta-analysis. J Am Coll Cardiol.

